# Pdgfrα functions in endothelial-derived cells to regulate neural crest cells and the development of the great arteries

**DOI:** 10.1242/dmm.029710

**Published:** 2017-09-01

**Authors:** Haig Aghajanian, Young Kuk Cho, Nicholas W. Rizer, Qiaohong Wang, Li Li, Karl Degenhardt, Rajan Jain

**Affiliations:** 1 Departments of Medicine and Cell and Developmental Biology, Penn Cardiovascular Institute, Institute for Regenerative Medicine, Perelman School of Medicine at the University of Pennsylvania, Philadelphia, PA 19104, USA; 2 Department of Pediatrics, Chonnam National University Medical School, Gwangju, 61186, South Korea; 3 Children's Hospital of Philadelphia, University of Pennsylvania, Philadelphia, PA 19104, USA

**Keywords:** Pdgfrα, Cardiogenesis, Neural crest, Endothelium, Outflow tract

## Abstract

Originating as a single vessel emerging from the embryonic heart, the truncus arteriosus must septate and remodel into the aorta and pulmonary artery to support postnatal life. Defective remodeling or septation leads to abnormalities collectively known as conotruncal defects, which are associated with significant mortality and morbidity. Multiple populations of cells must interact to coordinate outflow tract remodeling, and the cardiac neural crest has emerged as particularly important during this process. Abnormalities in the cardiac neural crest have been implicated in the pathogenesis of multiple conotruncal defects, including persistent truncus arteriosus, double outlet right ventricle and tetralogy of Fallot. However, the role of the neural crest in the pathogenesis of another conotruncal abnormality, transposition of the great arteries, is less well understood. In this report, we demonstrate an unexpected role of *Pdgfra* in endothelial cells and their derivatives during outflow tract development. Loss of *Pdgfra* in endothelium and endothelial-derived cells results in double outlet right ventricle and transposition of the great arteries. Our data suggest that loss of *Pdgfra* in endothelial-derived mesenchyme in the outflow tract endocardial cushions leads to a secondary defect in neural crest migration during development.

## Abstract (precis)

**Summary:** Loss of Pdgfrα in endothelial-derived mesenchyme results in defective neural crest behavior and is associated with conotruncal defects including, surprisingly, transposition of the great arteries.

## INTRODUCTION

The cardiac truncus arteriosus is the embryonic precursor to the great arteries, the ascending aorta and the pulmonary artery. The ascending aorta will connect the left ventricle to the systemic circulation, while the pulmonary artery will connect the right ventricle to the pulmonary circulation. A complex process of remodeling occurs to the outflow tract (OFT) during development, and perturbations to this process that lead to congenital heart defects are defined as conotruncal anomalies (Neeb et al., 2013; Restivo et al., 2006). Persistent truncus arteriosus (PTA) describes a morphological defect in which outflow tract fails to septate into two discrete arteries. Double outlet right ventricle (DORV) is morphologically characterized by both the aorta and pulmonary artery arising from the right ventricle. Tetralogy of Fallot (ToF) is classically characterized by a constellation of four abnormalities: an overriding aorta, ventricular septal defect, pulmonary valve defects and right ventricle hypertrophy. Finally, transposition of the great arteries (TGA) describes a condition in which the aorta incorrectly connects to the right ventricle and the pulmonary artery incorrectly connects to the left ventricle. Many conotruncal abnormalities are accompanied by a ventricular septal defect that allows shunting of blood between ventricles. However, when uncorrected, these abnormalities result in circulation of mixed deoxygenated and oxygenated blood, which is associated with severe morbidity and mortality. All together, conotruncal anomalies account for a significant proportion of all congenital heart defects.

Multiple lineages coalesce and interact during the development of the outflow tract, which begins as an endothelial-lined myocardial tube that will rotate, septate and ultimately give rise to a discrete aorta and pulmonary artery (Kirby, 2007; Snarr et al., 2008). Decades of research have shown that local signals exchanged between endothelium, neural crest and myocardial progenitor cells in the outflow tract cushions regulate outflow tract remodeling. A subset of endothelial cells will undergo endothelial-to-mesenchymal transition (EMT) to populate the outflow tract cushions. The neural crest is a multipotent group of ectodermally derived cells that give rise to neurons, melanocytes, adrenal medulla, dorsal root ganglia, and craniofacial cartilage and bones (Engleka et al., 2005; Lang et al., 2005). Cardiac neural crest cells migrate from the neural tube through pharyngeal mesoderm and populate the outflow tract cushions during midgestation (Engleka et al., 2005; Jiang et al., 2000; Kirby et al., 1983). These cells will eventually differentiate into the smooth muscle surrounding the proximal pulmonary artery and ascending aorta, and contribute to semilunar valve remodeling. Classical studies that removed the cardiac neural crest in chick or genetically ablated crucial determinants of neural crest in murine embryos result in many conotruncal abnormalities, including PTA and DORV, indicating their central role in remodeling the outflow tract (Epstein et al., 1993; Franz, 1989; Kirby et al., 1983, 1985; Morgan et al., 2008; Olaopa et al., 2011). More recent studies have demonstrated a role for neural crest in semilunar valve remodeling, providing a cellular link for individuals with bicuspid aortic valve and aortopathies (Jain et al., 2011). These studies demonstrated that neural crest cells provide an inductive signal to mediate late-gestation semilunar valve remodeling and apoptosis. Loss of neural crest migration resulted in abnormally large valves. Finally, second heart-field progenitor cells marked by islet1 expression migrate from the pharyngeal mesoderm into the outflow tract to give rise to myocytes (Cai et al., 2003; Jain et al., 2015). Progenitor cells commit to the myocyte lineage in the outflow tract, and provide local signals to guide neural crest migration (McCulley et al., 2008; Ward et al., 2005).

The molecular determinants of cellular interaction during cardiac development are of intense interest. Platelet-derived growth factor (Pdgf) family members have been shown to regulate outflow tract development (Tallquist and Soriano, 2003). Two receptors and four ligands can homodimerize and heterodimerize in several combinations and make up the family of Pdgfs (Fredriksson et al., 2004). Activation of the receptor tyrosine kinase requires binding of a dimer of ligands and results in a variety cell-specific responses, including cellular proliferation, differentiation and cell migration (Fredriksson et al., 2004). A *de novo* mutation in the murine *Pdgfra* locus (*Patched*) results in multiple phenotypes associated with abnormalities in neural crest, including craniofacial and outflow tract defects (Gruneberg and Truslove, 1960; Morrison-Graham et al., 1992; Smith et al., 1991; Stephenson et al., 1991). This result catalyzed several studies investigating the role of individual components of the signaling pathway in neural crest, confirming the central role of Pdgf family members in regulating neural crest cells (Richarte et al., 2007; Tallquist and Soriano, 2003). However, the expression and role of Pdgf signaling in non-neural crest lineages that contribute to outflow tract development has been less well studied.

In this report, we demonstrate an unexpected role for *Pdgfra* in endothelial cells and their derivatives during outflow tract development. Loss of *Pdgfra* in endothelium and endothelial-derived cells results in conotruncal abnormalities, including DORV and TGA, characterized by hypocellular outflow tract cushions. Our data demonstrate that *Pdgfra* is highly expressed in mesenchyme derived from endothelial cells that is in close proximity to migrating neural crest. Finally, we demonstrate that loss of *Pdgfra* in endothelial-derived cells leads to a defect in neural crest migration.

## RESULTS

### *Pdgfra* deletion in endothelium results in outflow tract defects

**Fig. 1.**
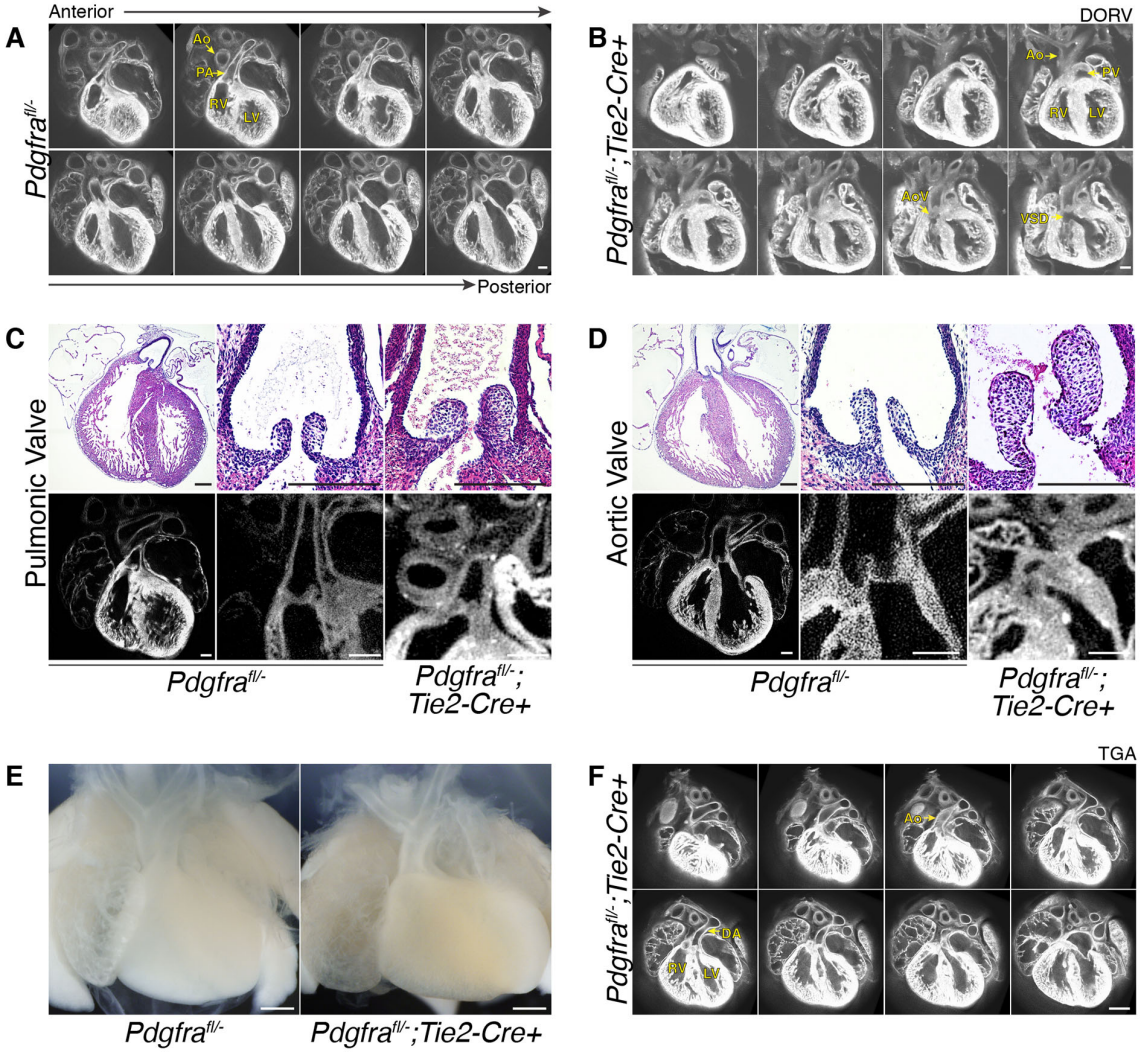
**Pdgfrα regulates outflow tract development.** (A,B) Serial images from optical projection tomography from either control (*Pdgfra^fl/−^*) (A) or mutant (*Pdgfra^fl/−^*;*Tie2-Cre*+) (B) late gestation hearts, which demonstrate DORV with VSD. (C,D) Histology (coronal sections) and optical projection tomography images of semilunar valves from control (*Pdgfra^fl/−^*) (C) or mutant (*Pdgfra^fl/−^*;*Tie2-Cre+*) (D) valves. (E) Whole-mount bright-field image of control (*Pdgfra^fl/−^*) or mutant (*Pdgfra^fl/−^*;*Tie2-Cre+*) heart, great arteries and lungs. The mutant displays TGA. (F) Serial optical projection tomography images demonstrating TGA in mutant heart. Embryo shown in B is from a different litter from those shown in the rest of the images. Ao, aorta; PA, pulmonary artery; RV, right ventricle; LV, left ventricle; AoV, aortic valve; PV, pulmonic valve; VSD, ventricular septal defect; DA, ductal arch. All embryos are E17.5-E18.5. Scale bars: 200 µm in A-D,F; 500 µm in E.

To examine the role of platelet-derived growth factor receptor alpha (Pdgfrα) in developmental endothelium, we crossed *Pdgfra^+/−^;Tie2-Cre+* males to *Pdgfra^fl/fl^* females to conditionally delete *Pdgfra* in endothelial cells and their derivatives. The floxed (*Pdgfra^fl^*) (Tallquist and Soriano, 2003) and null (*Pdgfra^−^*) (Hamilton et al., 2003) alleles have been previously generated and characterized. No mutant pups were found alive, and hence we analyzed late gestation embryos harvested by Cesarean section or born under direct observation. To our surprise, 6/15 *Pdgfra^fl/−^;Tie2-Cre+* mutants demonstrated structural outflow tract defects. Using optical projection tomography (Sharpe et al., 2002), we found three out of 15 late gestation mutants (E17.5-P0) in which the aorta and pulmonary artery emanate from the right ventricle in conjunction with a ventricular septal defect (DORV) (Fig. 1A,B; Movie 1, control *Pdgfra^fl/−^*; Movie 2, mutant *Pdgfra^fl/−^;Tie2-Cre+*). Two of these mutants also displayed an atrioventricular canal defect (Fig. S1). No outflow tract abnormalities were found in littermate, age-matched control embryos (over 60 control embryos examined, Fig. 1A, Table 1; Movie 1). In embryos that did not have an apparent structural OFT defect, dysmorphic semilunar valve leaflets were frequently observed, which have been previously associated with neural crest abnormalities (Fig. 1C,D). Mutant aortic valves were 2.2 times larger than control [346±59 arbitrary units (a.u.) versus 766±129 a.u., *n*≥3, *P*<0.05] and mutant pulmonic valves were 1.6 times larger than control (313±38 a.u. versus 491±18 a.u., *n*≥3, *P*<0.05).

**Table 1.**
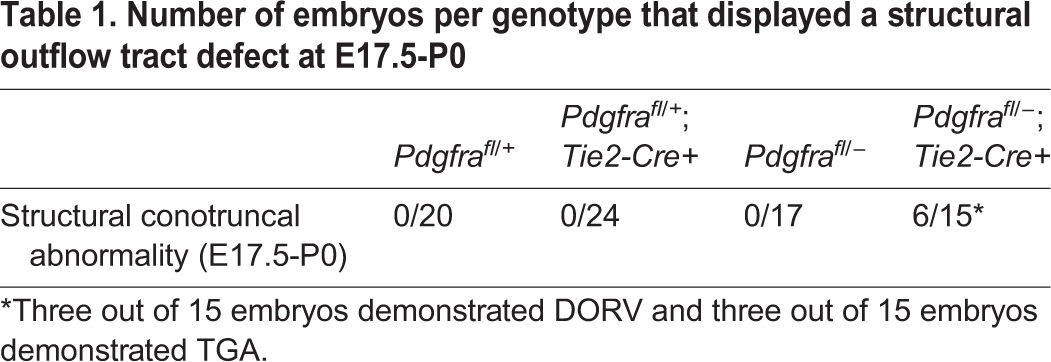
**Number of embryos per genotype that displayed a structural outflow tract defect at E17.5-P0**

Most strikingly, we observed TGA in three out of the 15 late gestation embryos (Fig. 1E; Movie 3; Table 1). In these embryos, the right ventricle aligned to the aorta and gave rise to the systemic circulation, whereas the pulmonary artery aligned with the left ventricular outflow tract. We did not detect discordance between the atria and ventricles, but we did detect ventricular septal defects in each mutant. TGA was not observed in control littermate embryos (Table 1). We confirmed misalignment of outflow tracts using optical projection tomography (Fig. 1F). Interestingly, *Pdgfra^fl/fl^;Tie2-Cre* embryos did not show apparent outflow tract abnormalities or valve defects.

### Endothelial-derived cells express Pdgfrα in the outflow tract

**Fig. 2.**
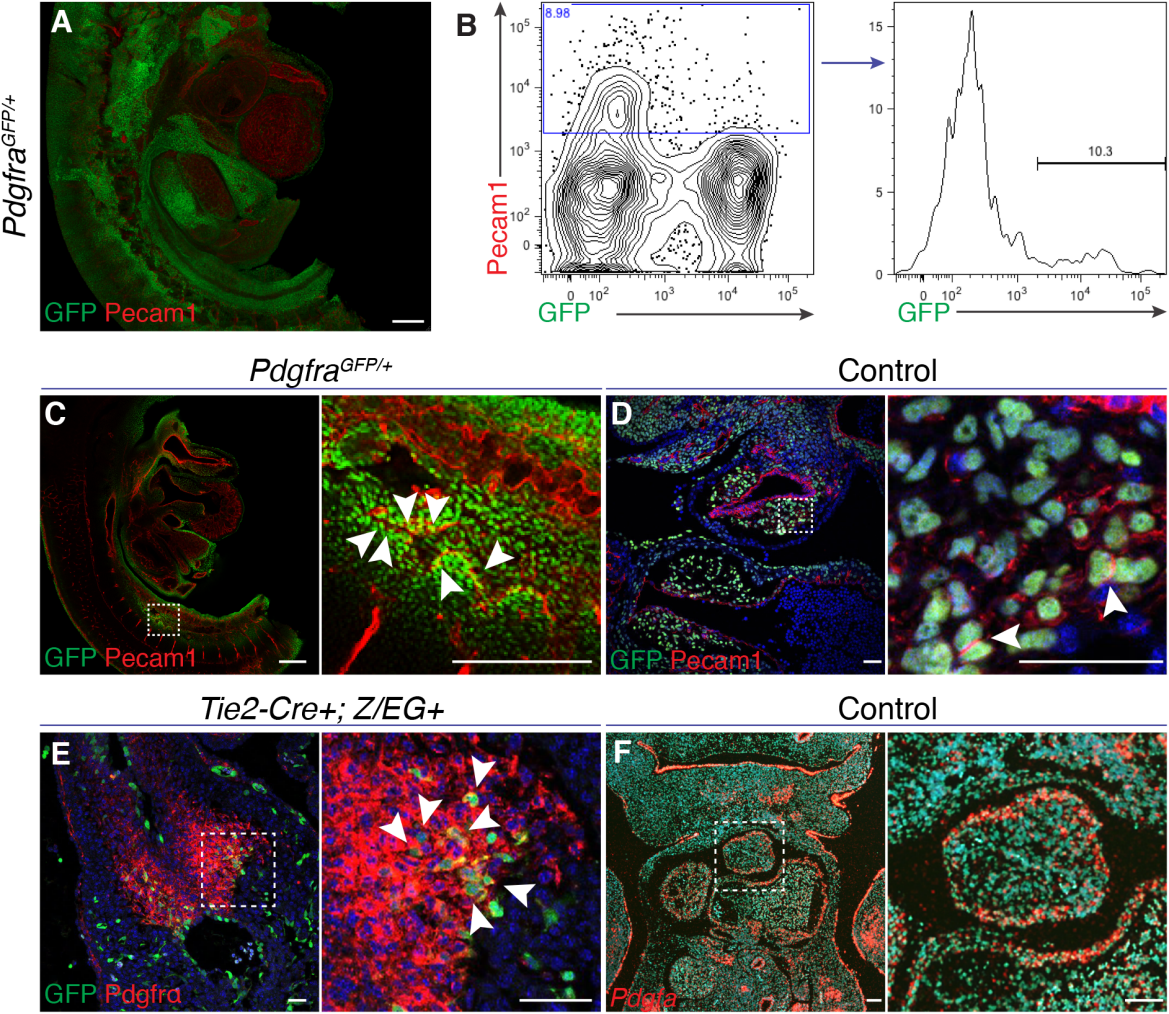
**Pdgfrα is expressed in endothelial-derived mesenchyme.** (A) Reconstruction of confocal images of whole-mount immunostaining of Pdgfra (GFP, green) and Pecam1 (red) in a E11.5 *Pdgfra^GFP/+^* embryo. (B) Flow cytometry plots from E11.5 embryos demonstrating the population of double-positive Pdgfrα^+^, Pecam1^+^ cells. (C) Sagittal confocal image demonstrating Pecam1^+^ (red) Pdgfrα^+^ (green) cells in the proximity of the dorsal aorta (white arrowheads). (D) Coronal section of E11.5 *Pdgfra^fl/−^* embryo demonstrating Pecam1^+^ (red) Pdgfrα^+^ (green) cells in the outflow tract cushion (highlighted by white arrowheads). (E) Tie2 lineage-traced cells (green) express Pdgfrα (red) in the outflow tract cushions (E12.5, white arrowheads, cross-section). (F) *In situ* hybridization images of *Pdgfa* expression in coronal section of outflow tract (E10.5) of *Pdgfra^fl/+^;Tie2-Cre+* embryo. In C-F, the boxed areas are shown at higher magnification on the right. Scale bars: 50 µm in D,E; 100 µm in F; 250 µm in A,C.

Shortly after looping of the linear heart tube, the conotruncal cushions develop and begin to remodel (E10.5-E11.5). Many outflow tract defects have their origins in abnormal cushion development or maturation. Therefore, we hypothesized that the endothelium overlying the outflow tract cushions at E11.5 expresses Pdgfrα. We used whole-mount imaging and flow cytometry to identify Pdgfrα^+^ endothelial cells (Fig. 2A,B; Movie 4). We generated single cell suspensions from E11.5 *Pdgfra^GFP/+^* embryos and used flow cytometry to define a small proportion of Pecam1^+^ cells which were GFP^+^ (10.3%, Fig. 2B). Upon close examination of whole mount embryos, we found Pdgfrα^+^ Pecam1^+^ endothelium in the dorsal aorta and surrounding blood vessels (Fig. 2C). We did not identify Pdgfrα^+^ endothelium overlying the outflow tract cushions. However, Pdgfrα is widely expressed in the outflow tract cushion mesenchyme at E11.5 (Fig. 2D). Endothelial cells undergo EMT and populate the outflow tract cushion mesenchyme. Therefore, we lineage traced endothelial cells by crossing *Tie2-Cre+* mice to mice harboring a Cre-dependent reporter allele (*Z/EG*). At E11.5-E12.5, we found that both endothelial-derived and non-endothelial-derived cushion mesenchyme express Pdgfrα (Fig. 2E; Fig. S2A). Consistent with previous reports (Tallquist and Soriano, 2003), we also detected that neural crest-derived mesenchyme and adjacent non-neural crest-derived cushion mesenchyme express Pdgfrα (Fig. S2B), suggesting the OFT may be an area of reciprocal signaling between the endothelial-derived mesenchyme and neural crest. Additionally, in contrast to the developing chick, in which *Pdgfra* is expressed in the myocardial cuff of the outflow tract and platelet derived growth factor A (*Pdgfa*) is expressed in the cushion mesenchyme (Van Den Akker et al., 2005), we detected *Pdgfra* expression in the cushion mesenchyme and *Pdgfa* ligand in the outflow tract myocardium of E11.5 embryos (Fig. 2F). Taken together, our data demonstrate that Pdgfrα is expressed in scattered endothelial cells in the dorsal aorta and that it is widely expressed by endothelial-derived mesenchyme in the outflow tract cushions of mid-gestation embryos.

### Pdgfrα regulates outflow tract cushion development

**Fig. 3.**
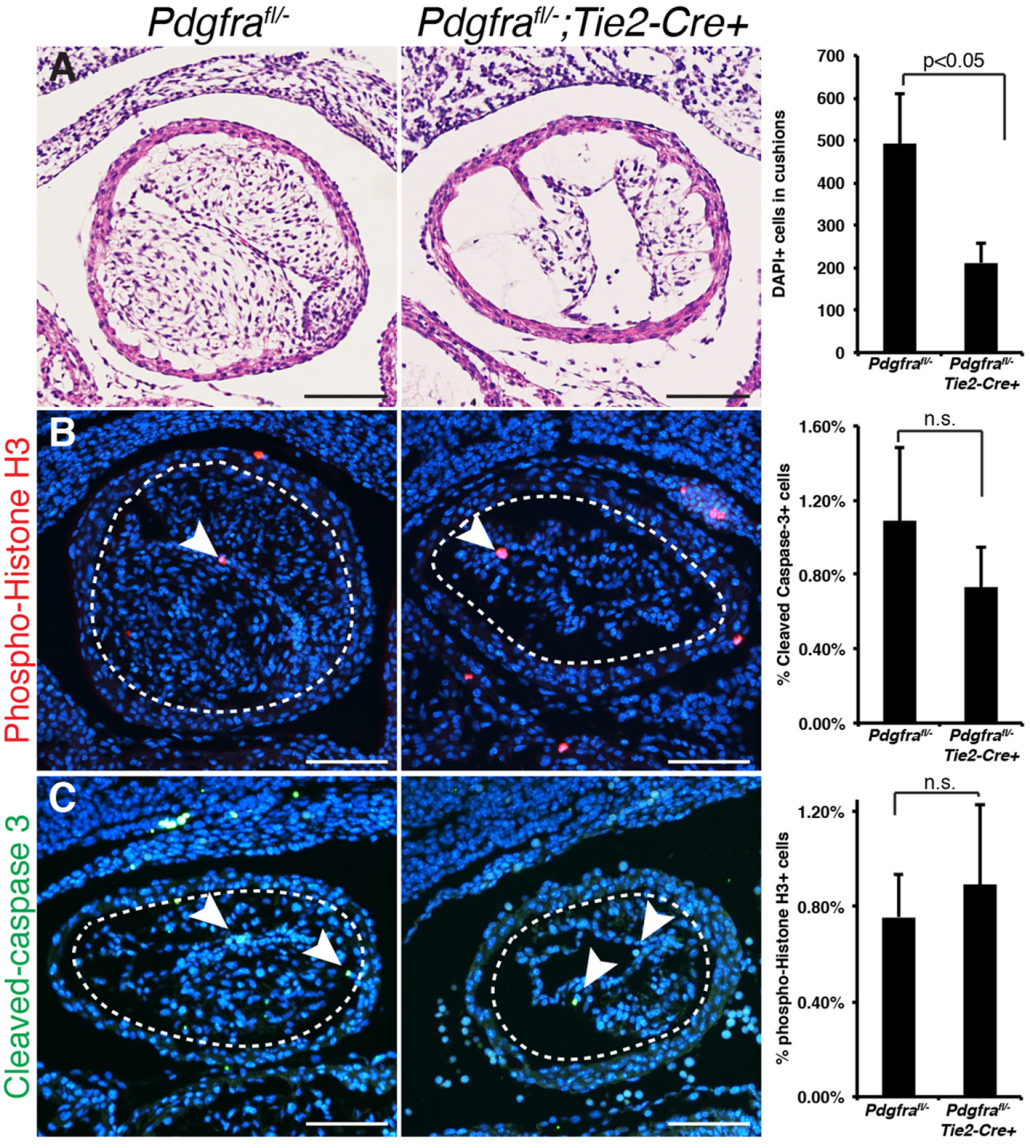
**Loss of Pdgfrα in endothelium and derived cells results in hypocellular outflow tract cushions.** (A) Histology of control (*Pdgfra^fl/−^*) and mutant (*Pdgfra^fl/−^*;*Tie2-Cre+*) outflow tract cushion coronal sections, and quantitation of cellularity by DAPI^+^ cells in cushions. (B,C) Representative images and corresponding quantitation of phospho-histone H3^+^ (red, arrowheads) (B) and cleaved caspase 3^+^ (green, arrowheads) (C) cells in coronal sections of outflow tract cushions, normalized to number DAPI^+^ (blue) cells in outflow tract cushion. The area quantified is indicated by a dotted circle, and cells in the surrounding myocardial cuff were excluded from our analysis. In all cases, *Pdgfra^fl/−^* was used as control; *n*≥3 E11.5-12.5 embryos (see Materials and Methods). Data are mean±s.e.m., n.s. indicates not significant (two-tailed *t*-test). Scale bars: 100 µm.

Given the expression pattern of Pdgfrα and phenotypes observed, we analyzed the outflow tract cushions from E11.5 control (*Pdgfra^fl/−^*) and mutant (*Pdgfra^fl/−^;Tie2-Cre+*) embryos. Mutant outflow tract cushions were hypocellular when compared with control (Fig. 3A). In addition, rates of apoptosis and proliferation, based on cleaved caspase 3 and phospho-histone H3 immunohistochemistry, respectively, were similar among cells residing in the outflow tract cushion mesenchyme in control and mutant embryos (Fig. 3B,C). We assessed rates of proliferation and apoptosis (relative to DAPI^+^ cells) in the OFT mesenchyme and immediately adjacent pharyngeal mesoderm in E10.5 control (*Pdgfra^fl/−^*) or mutant (*Pdgfra^fl/−^;Tie2-Cre+*) embryos, and did not detect any statistically significant differences [proliferation: 0.9±0.3% versus 1.5±0.5% phospho-histone H3^+^ cells in control versus mutant, respectively, *P*=nonsignificant (n.s.), *n*≥3; apoptosis: 1.4±0.7% versus 1.3±0.6% cleaved-caspase 3^+^ cells in control versus mutant, respectively, *P*=n.s., *n*≥3]. The hypocellularity of the cushions, combined with the lack of a clear difference in proliferation and apoptosis, led us to hypothesize that cell populations might be missing in the outflow tracts of mutant embryos.

### Pdgfrα in endothelial-derived mesenchyme influences neural crest cells

**Fig. 4.**
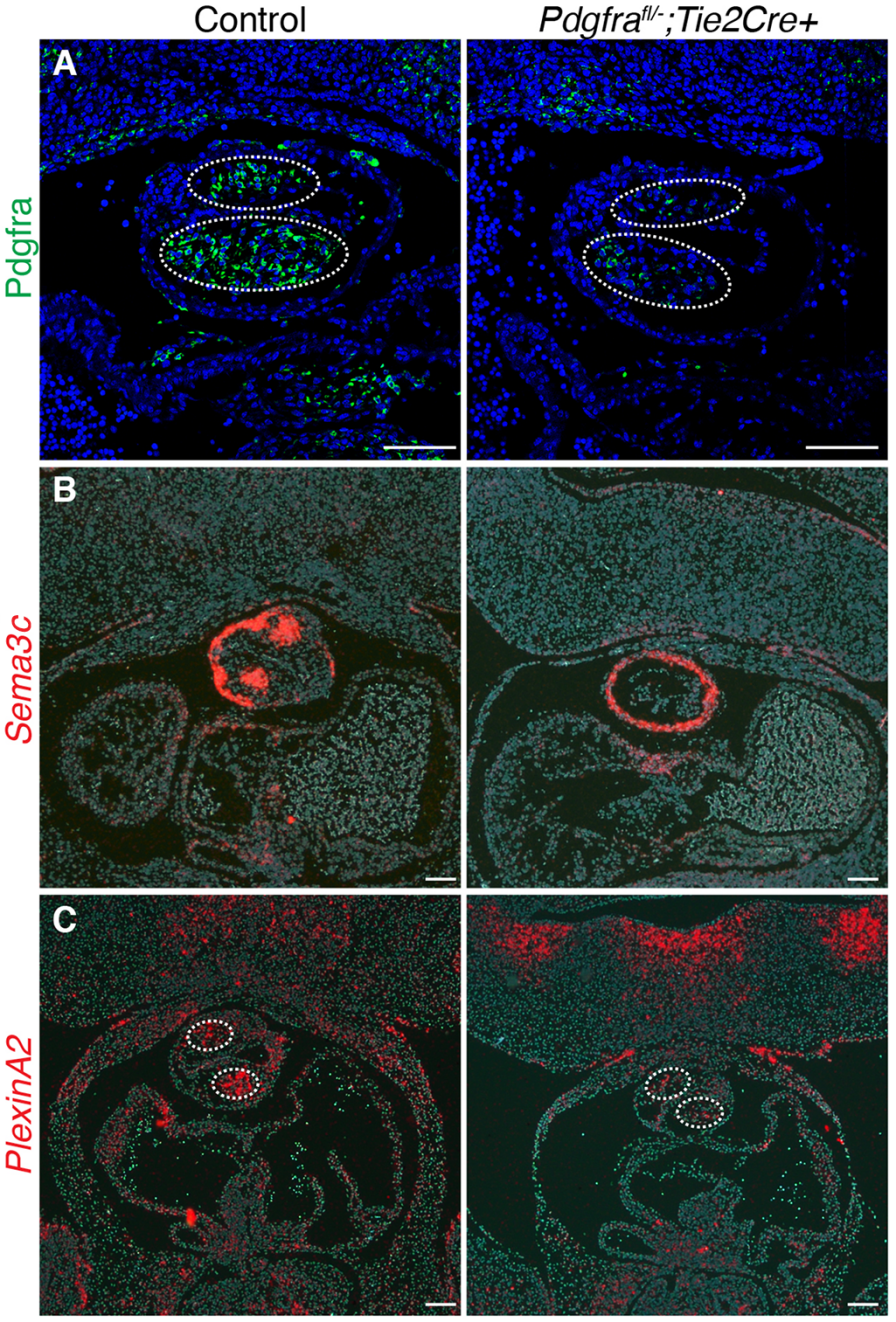
**Pdgfrα in endothelium and derived cells influences neural crest behavior.** (A) Pdgfrα staining (green) in the outflow tract of control (*Pdgfra^fl/−^*) versus a mutant embryo (*Pdgfra^fl/−^*;*Tie2-Cre+*). Pdgfrα expression is reduced in the mesenchyme of outflow tract cushions (dotted outline) in the mutant compared with the control. (B,C) *In situ* hybridization of *Sema3c* (B) and *Plxna2* (C, dotted oval) expression in the outflow tract cushions of control (*Pdgfra^fl/+^;Tie2-Cre*) and mutant (*Pdgfra^fl/−^*;*Tie2-Cre+*). All embryos were E11.5-12.5 and images are coronal sections. Scale bars: 100 µm.

Although Pdgfrα is expressed by both endothelial-derived (Fig. 2E) and non-endothelial-derived cushion mesenchyme (Fig. S2), we found a near complete absence of Pdgfrα in the outflow tract cushions of mutant embryos (Fig. 4A). We confirmed that endothelial cells were present lining the outflow tract cushions of mutant embryos, and did not detect a difference in Snail expression, a marker of endothelial-to-mesenchymal transformation, in the cushions of mutant versus control embryos (Fig. S3 and S4). Given the expression of Pdgfrα in both neural crest and endothelial-derived mesenchyme residing in the outflow tract (Fig. 2E; Fig. S2) and the near-complete loss of Pdgfrα expression in the outflow tract cushions in mutants, we hypothesized that neural crest cells may be missing from the mutant cushions.

Neural crest migrates into the outflow tract and invades the endocardial cushions as two tightly condensed prongs of cells that express *Sema3c* and plexin A2 (*Plxna2*) (High et al., 2009). We detected a paucity of cells expressing *Sema3c* or *Plxna2* in the outflow tracts of mutants using *in situ* hybridization (Fig. 4B,C). We observed 43.3±2.4% of cells of E11.5 OFT mesenchyme are *Sema3c*^+^ in control embryos (*Pdgfra^fl/+^;Tie2-Cre+*) versus 4.9±0.7% in mutant embryos (*Pdgfra^fl/−^;Tie2-Cre+*; *P*<0.001, *n*≥3). Additionally, we did not detect a statistically significant difference in number of non-*Sema3c*-expressing OFT mesenchymal cells between control and mutant (220.6±26.5 versus 191.7±17.4 DAPI^+^ cells, respectively, *P*=n.s., *n*≥3). We detected neural crest cells, marked by *Sema3c* expression or AP-2α (Tfap2a) expression (High et al., 2009; Park et al., 2008), in the pharyngeal mesoderm immediately posterior to the OFT in control and mutant embryos (Fig. S5), consistent with neural crest being able to migrate to the pharyngeal mesoderm in the mutant background. Furthermore, migrating neural crest cells contribute to multiple cell types in various tissues and organs in the body. *Pdgfra^fl/−^;Tie2-Cre+* embryos appear to have normal neural crest-derived structures when compared with controls, including the dorsal adrenal medulla, tooth primordia and dorsal root ganglia (Fig. S6). Taken together, these data suggest that Pdgfrα in the endothelium and derived cells is required for proper neural crest cell migration into the outflow tract cushions and subsequent semilunar valve (Jain et al., 2011) and outflow tract remodeling.

## DISCUSSION

Multiple cell types coalesce in the outflow tract cushions to remodel the primitive outflow tract into the great arteries (Kirby, 2007; Snarr et al., 2008). Abnormalities in any of the cell types results in phenotypes mimicking common congenital heart disease, and hence identifying factors that mediate interactions between cell types is of great interest. It is well established that Pdgfrα in neural crest cells influences outflow tract development (Richarte et al., 2007; Tallquist and Soriano, 2003). Our data demonstrate that disruption of Pdgfrα in endothelial cells and their derivatives results in conotruncal abnormalities, including DORV and TGA, in a subset of late gestation mutants. We detected Pdgfrα expression in the endothelial-derived mesenchyme, distinct from neural crest cells expressing Pdgfrα, in the outflow tract cushions. Finally, we identified a paucity of neural crest cells in the outflow tract cushions of mutant embryos. Taken together, our data suggests that Pdgfrα signaling in endothelial-derived mesenchyme influences neural crest behavior.

Though database analyses suggest Pdgfrα expression in endothelium in cell lines and tissues (e.g. www.ebi.ac.uk/gxa/home, http://biogps.org and www.proteinatlas.org), we are unaware of a functional characterization of Pdgfrα in endothelial cells. Our data, to the best of our knowledge, provide an initial description of a functional role for Pdgfrα in endothelial cells and their derivatives. Our expression analyses demonstrate that Pdgfrα is expressed in endothelium during development, including vessels emanating from the dorsal aorta. Based on the expression pattern of Pdgfrα in the outflow tract cushions, we suspect that Pdgfrα signaling is active in the endothelial derived mesenchyme. However, we cannot formally exclude a role in the endothelium overlying the cushion. In addition, although neural crest cells are present in the pharyngeal mesoderm immediately posterior to the heart in mutant embryos (Fig. S5), it is possible that earlier points of contact between the neural crest and endothelial lineages may additionally modulate neural crest behavior (Figs S5A, S7). Interestingly, we did not detect outflow tract defects in the *Pdgfra^fl/fl^;Tie2-Cre+* mutants. Our lineage-tracing results (Fig. 2E; Fig. S2A) suggest this may be secondary to efficiency of Cre recombinase expression in the endocardium at this developmental time point, but it is also possible that dampening Pdgfrα signaling in non-endothelial cells may sensitize embryos to endothelial-based phenotypes. Finally, our expression analyses suggest species differences. *Pdgfa* expression is localized to the cushion mesenchyme and Pdgfrα is expressed in the myocardial cuff in chick (Van Den Akker et al., 2005), whereas our studies show an inversion of this expression pattern in murine embryos.

Experiments that physically remove premigratory neural crest cells from chick embryos or genetic ablation experiments in murine embryos result in a wide array of outflow tract defects, including persistent truncus arteriorus, DORV and TGA (Epstein et al., 1993; Franz, 1989; Kirby et al., 1983, 1985; Morgan et al., 2008; Olaopa et al., 2011). More recently, neural crest defects have been linked to the pathogenesis of semilunar valve disease and associated aortopathies (Jain et al., 2011). However, it is less well understood whether TGA has a cellular etiology rooted in the neural crest (Unolt et al., 2013). Pioneering studies by Margaret Kirby and colleagues demonstrated TGA in 1/16 chick embryos in which neural crest cells were physically removed (Kirby et al., 1983). However, other studies manipulating neural crest cells recapitulated PTA and DORV, but failed to yield TGA (Epstein et al., 1993; Morgan et al., 2008; Olaopa et al., 2011). These conflicting results led to controversy around whether TGA was a primary neural crest defect or a defect of a related cell type. Our data demonstrate that neural crest migration and/or survival is abnormal in Pdgfrα-endothelial mutants and is associated with DORV and TGA. This raises the possibility that aberrant neural crest behavior is, at least in part, responsible for alignment of the great arteries over the correct ventricles. In addition, it suggests that some cases of TGA may be a malformation on the continuum of classic conotruncal abnormalities (Anderson et al., 1974). DiGeorge/velcardiofacial syndrome (del22q11) is thought to be characterized by abnormal neural crest behavior and presents with conotruncal abnormalities, including ToF, PTA or interrupted aortic arch B. However, intriguingly, a small fraction of individuals with del22q11 have presented with TGA (Van Mierop and Kutsche, 1986).

Abnormal myocardial rotation and spiraling of the great vessels have both been thought to contribute to the pathogenesis of TGA (de la Cruz et al., 1981; Goor and Edwards, 1973). Elegant studies by the Buckingham laboratory demonstrated the contribution of neural crest cells to myocardial rotation during outflow tract septation and alignment (Bajolle et al., 2006). Using a transgenic mouse line, which harbored an nLacZ cassette upstream of the *Myf5* locus, the group demonstrated that myocardium initially labeled on the right side of the outflow tract at E9.5 rotates over two days to the left and eventually becomes confined to the pulmonary trunk. The group went on to show that myocardial rotation is delayed in the well-studied Splotch mutants, which display DORV and PTA due to neural crest defects. *Pitx2c* mutants demonstrated similar myocardial rotation defects and a high penetrance of TGA. It was determined later that the aforementioned transgenic line harboring nLacZ is a reporter of *Sema3c* expression (Theveniau-Ruissy et al., 2008). Interestingly, our data demonstrate that *Sema3c* is expressed in approximately one-half of the myocardial cuff surrounding the OFT in control embryos. The *Sema3c* expression domain is expanded to encompass the entire myocardial cuff in the mutant (Fig. 4B). Therefore, it is possible that this mislocalization of *Sema3c* expression is consistent with a rotation defect as detected in Splotch, *Pitx2c* and *Tbx1* mutants (Bajolle et al., 2006; Theveniau-Ruissy et al., 2008), although further tools will be required to fully explore this possibility. As the molecular mechanisms and determinants of myocardial rotation are elucidated, it will be important to understand how endothelial cell-derived mesenchyme influences neural crest-mediated myocardial rotation and/or septation, and whether these defects are common to at least some forms of DORV and TGA.

Previous data have also implicated perlecan in the pathogenesis of TGA (Costell et al., 2002). Perelecan is a heparin proteoglycan sulfate that stabilizes basement membranes and sequesters growth factors in combination with other extracellular matrix proteins. Re-examination of late-gestation global perlecan mutants demonstrated that the majority of them display TGA. It was hypothesized that abnormal neural crest resulted in TGA, although, paradoxical to our results, hypercellularity of the outflow tract cushions was noted. Although we did not detect a difference in perlecan expression in E11.5 outflow tracts in our mutants when compared with controls (data not shown), it is possible that misexpression of perlecan or other extracellular matrix components may affect neural crest migration. Misexpression of extracellular matrix components has been linked to outflow tract defects (Lockhart et al., 2011). The hypocellularity demonstrated in the Pdgfrα mutant outflow tracts (Fig. 3A) may be accompanied by a change in extracellular matrix production, and it will be interesting to determine whether any change in extracellular matrix is causal or a by-product of abnormal neural crest migration. It is likely that precise migration and quantity of neural crest is needed for appropriate outflow tract septation and rotation, and abnormal proliferation or migration, whether too fast or too slow, may result in conotruncal abnormalities. Tissue-specific knockouts of perlecan will undoubtedly shed additional light on the cell-autonomous nature of this defect, and whether the phenotype observed is secondary to abnormal neural crest behavior.

It has become clear that a rich interplay between cell types exists over time and space to remodel the primitive outflow tract into the great arteries supporting proper circulation. Defining the exact signaling pathways that mediate these interactions will be paramount to further understanding normal and pathological development of the outflow tract. Multiple pathways have been implicated in outflow tract development, including Notch, Fgf and Bmp (Kirby, 2007; McCulley et al., 2008; Park et al., 2008; Zaidi and Brueckner, 2017; Zhang et al., 2008). Additional studies will address whether loss of Pdgfrα in endothelial-derived mesenchyme leads to dysregulation of these pathways in relevant cell types. Little is known about the identity of local signals generated in mesenchymal cells that influence neural crest migration. It will be of interest to determine whether published endothelial-specific mutants that display conotruncal abnormalities also demonstrate alterations in outflow tract mesenchyme, outflow tract rotation and/or abnormal neural crest migration. Our studies reveal an unexpected role for Pdgfrα in endothelial-derived mesenchyme, and suggest that TGA may be a result of abnormal neural crest behavior. As additional cellular and molecular determinants of tissue crosstalk are identified, a clearer picture will develop of the similarities and differences of various conotruncal abnormalities.

## MATERIALS AND METHODS

### Mice

*Pdgfra^fl^* (Tallquist and Soriano, 2003), *Pdgfra^−^* (Hamilton et al., 2003), *Wnt1-C*re (Jiang et al., 2000), *R26^Tom/+^* (Madisen et al., 2010), *Z/EG* (Novak et al., 2000) and *ROSA^mT/mG^* (Muzumdar et al., 2007) mice were obtained from The Jackson Laboratory (www.jax.org, 006492, 007669, 022137, 007908, 003920 and 007576, respectively). *Tie2-Cre* mice have been previously described (Kisanuki et al., 2001). All animal protocols were approved by the University of Pennsylvania Institutional Animal Care and Use Committee (IACUC).

### Histology, immunohistochemistry and *in situ* hybridization

Samples were fixed overnight (mid-gestation embryos) or for 48 h (E16.5-P0 embryos) with 2-4% paraformaldehyde and dehydrated through an ethanol series. Samples were then embedded in paraffin wax and sectioned. Antibodies used for immunostaining were mouse anti-AP2α (5E4, Developmental Studies Hybridoma Bank; 1:25), goat anti-GFP (3470, Cell Signaling; 1:100), chicken anti-GFP (GFP-1020, Aves; 1:500), rabbit anti-phospho-histone H3 (9701; Cell Signaling Technology; 1:20), mouse anti-phospho-histone H3 (9706; Cell Signaling Technology; 1:200), rabbit anti-cleaved caspase 3 (9664, Cell Signaling; 1:50), goat anti-Pdgfrα (AF1062, R&D Systems; 1:25-1:50)*,* rabbit anti-Pdgfrα (3164, Cell Signaling; 1:50), rabbit anti-RFP (600-401-379, Rockland; 1:50-1:250) and rat anti-Pecam1 (DIA-310, HistoBioTec; 1:20). Radioactive *in situ* hybridization was performed using previously described probes for *Sema3c* and *Plxna2* (High et al., 2009). Hematoxylin and Eosin-stained images were prepared using a standard staining protocol. Whole-mount staining was performed with indicated antibodies as previously described (Degenhardt et al., 2013). Immunohistochemistry and *in situ* hybridization images were analyzed using Adobe Photoshop. Brightness and contrast were altered identically in control and mutant images in all instances using Adobe Photoshop. Littermates were used as control images, unless otherwise indicated. Additional details of antibodies, concentrations used, histology and staining protocols can be provided upon request and also found at: www.pennmedicine.org/departments-and-centers/penn-cardiovascular-institute/core-facilities/histology-and-gene-expression-core. Valve leaflets and outflow tract mesenchyme (excluding myocardial cuff) were manually outlined and then the area and number of cells (DAPI^+^, cleaved-caspase 3^+^ and phospho-histone H3^+^), respectively, were quantified using ImageJ. The size of two or three leaflets per aortic or pulmonic valve were measured and averaged to define the size of the respective valve per animal. At least three aortic or pulmonic valves were averaged in each genotype to compare valve size. Features within ImageJ that quantitate area and count cells were used. The average number of *Sema3c*^+^ and DAPI^+^ cells was quantified from coronal sections of E11.5 OFT cushions from control or mutant embryos using ImageJ. At least three embryos were quantitated in each cohort (control or mutant). Immunohistochemistry was imaged on either a Nikon Eclipse 80i fluorescence microscope or Leica TCS SP8 confocal microscope. Embryos and isolated hearts were imaged in whole mount on an Olympus MVX10 stereomicroscope.

### Optical projection tomography

Embryos were harvested into ice-cold PBS, and the heart and great arteries were dissected. The organs were fixed overnight in 4% paraformaldehyde. Organs were embedded in 1% low-melt agarose, dehydrated in methanol and then cleared in 1:2 (v/v) benzyl alcohol and benzyl benzoate (Jain et al., 2011; Sharpe et al., 2002). Organs were then scanned using the Bioptonics OPT Scanner (3001 M). Image stacks were reconstructed using OsiriX software. Movies were generated using iMovie.

### Flow cytometry

Single cell suspensions were made from E11.5 embryos and filtered through 70 μm screens. Cells were incubated with anti-Pecam1 (557355, BD Biosciences; 1:100) for 30 min, washed and incubated with fluorescent secondary antibody. Cells were counted by flow cytometry and analyzed using Flowjo software (www.flowjo.com).

### Statistics

All data are presented as mean±s.e.m. unless otherwise noted. Two-tailed Student's *t*-test was used for comparison between two datasets. *P*<0.05 was considered to be statistically significant.
